# Suicide and Mortality in Individuals with Gambling Disorder and Matched Case controls – A Swedish Nationwide Register Study

**DOI:** 10.1007/s10899-025-10415-w

**Published:** 2025-07-29

**Authors:** Anna Karlsson, Marianne Balem, Helena Hansson, Anders Håkansson

**Affiliations:** 1 Department of Clinical Sciences Lund, Psychiatry, Faculty of Medicine, Lund University, Lund, Sweden; 2 Department of Pediatrics, Skåne University Hospital, Malmö, Sweden; 3 Université de Nantes, CHU Nantes, CHU Tours, INSERM, Methods in Patients centered outcomes and Health Research, SPHERE, Nantes, F-44000 France; 4 Nantes Université, CHU Nantes, UIC Psychiatrie et Santé Mentale, Nantes, F-44000 France; 5 Faculty of Social Sciences, School of Social Work, Lund University, Lund, Sweden; 6 Malmö Addiction Center, Malmö, Region Skåne Sweden

## Abstract

This study aimed to investigate suicide and general mortality in individuals with gambling disorder and to evaluate the effect of gambling disorder on mortality and death from suicide. This is a Swedish nationwide case-control, register based study with a ratio of 1:2. Controls were matched on gender, age and municipality. Cases were defined as all adults with a diagnosis of gambling disorder in Swedish in-patient and/or specialized out-patient health care 2005–2019. The total population included 10,782 individuals. Multifactor Cox regression stratified for sex was used to analyze risk factors for suicide mortality and general mortality. Suicide mortality was higher amongst cases, with 41 (1.2%) individuals passing away due to suicide vs. 22 (0.3%) of the controls (*p* < 0.001). In the regression model, gambling disorder was not significantly associated with suicide mortality, which was associated with substance use disorder and low education in men and for women the model could not draw conclusions on predicting factors. Mortality was also elevated in cases; 94 of the deceased were controls (1.3%) and 132 were cases (3.8%, *p* < 0.001). In the regression model gambling disorder was not significantly associated with mortality, this was predicted by socioeconomic status, increasing age, low education level, somatic comorbidity, substance use disorder and previous intentional self-harm in men and for women by increasing age and somatic comorbidity. In conclusion, gambling disorder is associated with increased mortality and suicide death. Comorbid disorders and socioeconomic status appear to be important reasons for the increased mortality.

## Background

Gambling disorder (GD) is a public health issue which appears to be associated with increased mortality and suicide mortality although knowledge is scarce. As GD is increasingly being recognized in healthcare and social services, knowledge on the risk of suicide and general mortality is much needed to provide relevant treatment.

GD is an addictive disorder with a pattern of “chasing losses” (where increased gambling is utilized in an attempt to regain lost money), gambling for increasing amounts, lying in order to hide gambling and continued gambling despite negative consequences.(*Diagnostic and statistical manual of mental disorders (5th ed.)*, 2013) Some individuals experience negative effects of gambling but do not fully meet the criteria of an addiction, and are referred to as problem gamblers.

Problem gambling affects around 5% of the population, (Calado & Griffiths, 2016) whereas lifetime prevalence of GD is estimated to around 0.5%. (Kessler et al., 2008; Petry et al., 2005) Although psychiatric comorbidity and suicidality is common in GD, many remain undiagnosed and the treatment uptake appears low, only around 10% (Shaffer & Korn, 2002) Prevalence of GD and problem gambling is higher in men than women (Calado & Griffiths, 2016; Hakansson et al., 2018); however, research from the past decades have demonstrated that gender differences in this regard are narrowing (Potenza et al., 2019), as the availability of gambling has favoured female gambling more than it did historically, sometimes referred to as a ‘feminization’ of gambling (Volberg, 2003; Svensson & Romild, 2014). In contrast to older findings, in recent years and in some research, among active gamblers, female gender even has emerged as a risk factor (Håkansson & Widinghoff, 2020). Psychiatric comorbidity is common in GD, and women are more often affected by comorbid disorders such as anxiety and depressive disorders (Bischof et al., 2015; Karlsson et al., 2021). Women also appear to develop gambling difficulties secondary to previous psychiatric comorbidity (Sundqvist & Wennberg, 2021). Further, economic hardship is common, and 45% of individuals with GD in Sweden had received social welfare payments within a follow up of 4.9 years (Karlsson et al., 2021).

A recent scoping review on suicide deaths related to problem gambling identified only 18 relevant articles, amongst which only a handful were original research articles, and concluded that literature on the subject is sparse (Mariya Andreeva, 2022). Most of the identified studies involved post mortem investigations such as examinations of coroner files or studies on interviews with near ones to the deceased (Mariya Andreeva, 2022).

A study on 44 coroner files related to gambling concluded that co-morbid depression, severe financial debt, relationship difficulties, introversion and low self-esteem might be risk factors for suicide death amongst problem gamblers and that the highest risk period may be following a serious loss, disclosure of criminal offenses or gambling-related debts (Blaszczynski & Farrell, 1998) Bourget et al. hypothesized after investigating 79 coroner files that the risk of suicide might be linked to multiple losses as a consequence of the problem gambling itself.(Bourget D, 2003) A study on 49 individuals deceased from suicide and 41 living individuals with at-risk or problem gambling, found deceased individuals to have had more mental health disorders during their last six months prior to death and to have experienced more financial difficulties (Andronicos et al., 2015) Séguin et al. found personality disorders to be twice as common in suicide victims with problem gambling, and that problem gamblers were much less likely to be in contact with health care prior to the suicide (Séguin et al., 2010). Wong and colleagues describe in an article on 17 suicide victims with GD that all had unmanageable debts, and that none had sought psychiatric health care, even though the majority also had depressive disorder (Wong et al., 2010). Another study from Wong et al., examining 1 201 coroner files, demonstrated a 19% incidence of prior gambling activities, among which 42% had been put into dept due to gambling (Wong et al., 2008). A previous nationwide register study on GD related suicide mortality appears to be the only study to estimate the risk of suicide death in individuals with GD compared to the general population with a 15-fold higher occurrence of suicide in gambling disorder patients (Karlsson & Hakansson, 2018; Mariya Andreeva, 2022).

Although research on suicide mortality and mortality in GD is scarce, more is known about suicidality and suicide attempts, which are also common in GD (Håkansson & Karlsson, 2020; Karlsson et al., 2021; Moghaddam et al., 2015; Ronzitti et al., 2017, 2018). Suicide attempts appear more abundant amongst women with GD (Bischof et al., 2015; Husky et al., 2015; Karlsson et al., 2021; Komoto, 2014; Pavarin et al., 2021). Several risk factors for suicide attempts have been described, including alcohol and drug use disorders, (Håkansson & Karlsson, 2020; Karlsson et al., 2021) depressive disorders, (Håkansson & Karlsson, 2020; Karlsson et al., 2021; Newman & Thompson, 2007) anxiety disorders, (Håkansson & Karlsson, 2020; Karlsson et al., 2021) personality disorders (Karlsson et al., 2021) such as cluster B personality disorder (as described in DSM-IV, (Association, 2000), and (Bischof et al., 2015) attention deficit disorder, (Retz et al., 2016) as well as female gender (Bischof et al., 2015; Håkansson & Karlsson, 2020; Husky et al., 2015). Further, a recent study indicates higher risks of suicide attempts within the first year of receiving a GD diagnosis within the health care system (Pavarin et al., 2021). GD often leads to economic hardship and indebtedness has been identified as a risk factor for suicidality in GD (Khazaal et al., 2017).

Few studies have carried out direct comparisons of suicide rates in gambling disorder patients in comparison to a control population. Thus, there is need for a more refined methodology, utilizing a case-control design.

The aim of the present study was to:

1. Compare mortality and suicide mortality levels and age of death between cases and controls.
2. Investigate the effect of gambling disorder on mortality and death from suicide compared to matched controls in relation to previously known risk factors.
3. Compare causes of death between cases and controls.

Our hypothesis being that controls would have increased mortality and suicide mortality as well as dying at a younger age. We further hypothesized that comorbid somatic disorders would be most important with regards to mortality levels and that GD would be a significant risk factor for suicide death.

## Methods

This is a nationwide case-control study. For the study population, all patients in Sweden with a GD diagnosis (F63.0, pathological gambling in ICD-10) between 2005 and 2019 were identified in the Swedish National Patient Register (*n* = 3689). Each case was paired, by the national authority Statistics Sweden (2005), with two age- and gender-matched controls living in the same municipality from the general Swedish population register. The matching was considered successful since two matching controls were found for every case and all cases and controls were unique. This resulted in a total study population of 11 067 with a follow-up from their individual entry into the study (e.g. date of first GD between 2005 and 2019 and the same date for the two matched controls) until censoring at 31 st of December 2019 or amongst those who deceased until date of death.

**Fig. 1:**
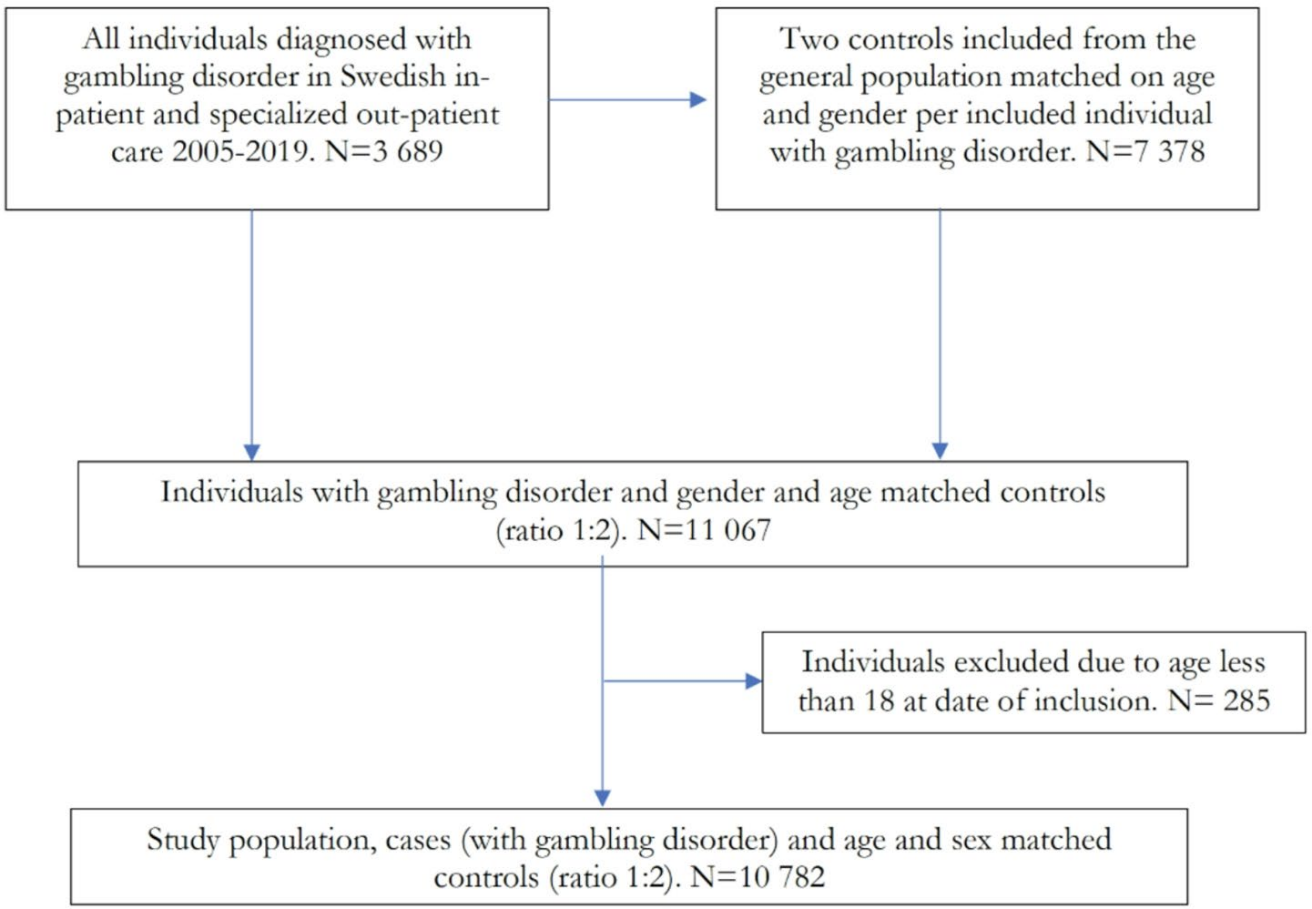
Flow chart on construction of the study population

Individuals younger than 18 years of age at inclusion were excluded (*n* = 285, 15 girls and 270 boys, with an age between 8 and 17). This was based on the assumption that up to recently, the active diagnosing of a GD in Swedish health care was believed to be very rare in minors. Therefore, given the very low number of minors appearing in the registers with a GD diagnosis even before this diagnosis became well-established in Swedish health care, it also cannot be excluded that some of these rare cases may have been children and adolescents who were mis-diagnosed due to extensive gaming habits not involving money (Håkansson et al., 2018). Thereafter, this resulted in a total study population of 10 782 cases and controls, with a 1:2 relationship. For these individuals, diagnoses were retrieved from the Swedish National Patient Register and the Swedish Cause of Death Register. Figure 1 depicts a flow chart of the study population.

*The Swedish National Patient Register* covers data on primary and secondary diagnoses according to the ICD-10 (the diagnostic manual utilized in national health care), although primary care is however not yet included in the register.(Welfare, 2018) The register is assessed to have a positive predictive value of 85–95% and covers 99% of all somatic and mental health hospital discharges and around 80% of all hospital-based outpatient care (Ludvigsson et al., 2011). Diagnoses retrieved for this particular study were substance use disorders, including alcohol use disorder (ICD codes F10-F19), depressive disorder (ICD F32-F33), anxiety disorder (ICD F40-F48), intentional self-harm (ICD X60-X84), diseases of the circulatory system (ICD chapter I), diseases of the respiratory system (ICD chapter J) and neoplastic disorders (ICD chapters C and D).

*The Swedish Cause of Death Register* is based on information provided from the death certificate in general determined by the treating physician. In the case of unclear deaths or deaths from injuries and accidents including suicide or deaths in individuals with known substance use, the report is instead provided by the forensic examiner and 95% of all deaths by suicide are registered in the Cause of Death Register.(Brooke et al., 2017) Diagnoses describing cause of death retrieved from the register are displayed in the left column in Table 3 and are based on ICD chapters for somatic causes and more in detail for causes of deaths evolving accident (ICD V0-X59), accidental poisoning (X40-X49), suicide (X60-X84), assault (X85-Y09, Y36-Y36) and events on undetermined intent (Y10-Y34).

*The Swedish Longitudinal integrated database for health insurance and labour market studies)* holds amongst others information on educational level, financial support, and unemployment. From this register information on unemployment, social welfare payments, sickness- activity- and rehabilitation compensation were retrieved and a binary variable on “socioeconomic vulnerability” was constructed. This factor was defined as any incidence of unemployment, social welfare payments, or sickness/activity/rehabilitation. There was no minimum limit for how long an individual had any of these factors (Larsson & Håkansson, 2022). However, sickness compensation requires that the individual is permanently unable to work, activity compensation requires an estimated sickness duration of at least one year, and rehabilitation compensation at least 60 days. Employment level is a category indicating an individual’s highest level of education (elementary school, high school, college and post-college education) from this variable we computed a dichotomous variable “elementary school education” dividing the material into those with elementary school (i.e. mandatory education until the 9th grade in Sweden) as their highest educational level and those with high school or further education. This division was chosen due to previous research indicating an increased suicide risk for those with highest education at elementary level, especially so for men (Phillips & Hempstead, 2017).

### Statistical Analyses

The statistical software program IBM SPSS Statistics 28 was used to process and analyze the data.

Age distribution was presented in means with standard errors. Pearson Chi-square analyses were conducted in order to investigate possible gender differences as well as potential differences between cases and controls with regards to death or death by suicide during follow-up. The independent samples t-test was utilized to compare age difference amongst cases and controls with regards to death in general and suicide death.

Causes of deaths were displayed for cases and controls separately and Chi square tests and Fischer’s tests were used to examine difference in prevalence between cases and controls. Benjamin Hochberg correction was used to correct significance level due to multiple testing.

Multifactor Cox regression analysis with general mortality as outcome were then conducted for men and women separately. In the model for men the factors included were case/control, GD, age respiratory, cardiac, malignant disease as well as substance use disorder, previous intentional self-harm, depression, anxiety disorder socioeconomic status and elementary school education. The variables respiratory, cardiac, malignant disorder as well as previous intentional self-harm (or suicide attempt) were chosen, since they were the leading causes of death. The variables substance use disorder, depression and anxiety were chosen due to their high prevalence rates amongst the cases and previous knowledge on their impact on mortality in previous research and were thus hypothesized to impact mortality rates. Socioeconomic status and educational level have also been considered to impact general mortality (Probst et al., 2014). For women, fewer variables were chosen due to the smaller size of the group; case/control, age, somatic comorbidity (with respiratory, cardiac, or malignant disease computed into a dichotomous variable 1/0), psychiatric comorbidity at large (ICD chapter F-diagnosis excluding F63.0, gambling disorder) and socioeconomic status were chosen for the multifactor Cox regression model.

A multifactor Cox regression analyses stratified for sex was then run with the outcome of suicide death as underlying or contributing cause of death (ICD X60-X84) for cases vs. controls to investigate the effect of gambling disorder on suicide death. A multivariate Cox regression was then conducted for men with case/control, substance use disorder, previous intentional self-harm, depression, and anxiety disorder, age, and education level as the independent variables. Variables were chosen due to their previously described impact on the risk of suicide death. For women a smaller model was constructed with factors: case vs. control, age and psychiatric comorbidity at large. The number of co-variates was kept lower in this analysis due to the lower level of suicide deaths in comparison to deaths in general.

Time in study was defined as the time between date of first GD for cases (and the same date for the two matched controls) and censoring; date of death or study end (December 31, 2019).

Information on date of death was incomplete for four individuals where only the year of death was stated. June 15 during that particular year was then chosen as their date of death.

The assumption of proportional hazards was a key consideration for all Cox regression models. We tested the proportional hazard assumption for our Cox Regression models with suicide mortality (X60-84) and general mortality separately with Kaplan Meyer curves (to visually inspect the proportional hazards assumption), and log(-log) plots for categorical and continuous variables, and for age, we used the Scale Schoenfeld Residual test (Kuitunen et al., 2021). As gender did not fit with the assumption of proportional hazards on the Kaplan Meyer curves and log(-log) plots the analyzes was stratified for gender. Interestingly, elementary school education proved to have a proportional hazard for men but not women. This analysis was dropped for women as not as many analyses could be performed due to the lower levels of mortality and suicide (Probst et al., 2014).

For each Cox regression model, model diagnostics were performed to assess the fit of the model, using Cox-Snell residuals and deviance residuals to evaluate the residual variance and the goodness-of-fit. Residuals were plotted to check for patterns suggesting a poor fit or violations of proportional hazards. A concordance index (C-index) was calculated for each model to assess the discriminatory power of the model in predicting mortality and suicide outcomes, using a 0.70 cut-off above which the predictive performance was considered acceptable. No variables in the models were centered or standardized. Standardization of socio-economic status was considered but not chosen, in order to avoid obscuring the interpretability of effects.

Results were reported in accordance with the STROBE checklist for case-control studies which can be found in appendix A. (von Elm et al., 2007). The study was however not pre-registered and results should thus be considered exploratory.

### Ethics

The study procedures were carried out in accordance with the Declaration of Helsinki, although consent was not obtained due to the nature of the registers. The study was approved by the Swedish Ethical Review Authority (Ethical approval number: 2019 − 01559).

## Results

The population consisted of 10 782 individuals, of whom 8 368 (77.6%) were men and 2 414 (22.4%) women. Age at inclusion ranged from 18 to 93 years of age with a mean of 36.3 years of age (standard error 0.11). The women were older, with a mean of 40.5 years of age (standard error 0.12), than the men, with a mean age of 35.1 years of age (standard error 0.11). Individuals were followed for a mean of 4.6 years (standard error 0.05). There was no difference in age at inclusion and proportion of women between the case and control group indicating successful matching.

During follow-up, 226 individuals passed away, of whom 94 were controls (1.3%) and 132 cases (3.8%), with a significantly higher percentage of cases deceasing (*p* < 0.001). This increased mortality was significant for both men and women, Table 1.

**Table 1:** Number and percentage of individuals passing away or passing away due to suicide during the study period, cases vs. controls with pearson Chi square comparison on death rate and suicide death, case vs. control separated for men and women. Fischers test was utilized for women with regards to suicide mortality indicated with: ^F^

		*N* of cases	% of cases	*N* of controls	% of controls	*p*-value, Pearson chi square test
General mortality	*Men	103	3.8	80	1.4	< 0.001
	*Women	29	3.6	14	0.9	< 0.001
Suicide mortality	*Men	36	1.3	18	0.3	< 0.001
	**Women**	5	0.6	4	0.2	0.165^F^

*Statistically significant at the 95% confidence level

Cases were generally younger when they deceased (*p* < 0.001) with a mean age of death for cases at 50.5 (standard error 1.27), compared to 57.7 (standard error 1.61) for controls. This difference was seen amongst men and women separately, Table 2.

**Table 2:** Mean age at death of individuals passing away during the study period from death in general and death from suicide. The independent samples t-test was conducted to compute differences on age of death, case vs. control separated for men and women. Displayed as mean age with a 95% confidence interval (CI)

		Age of death, cases	Standard error of the mean, cases	Age of death, controls	Standard error of the mean, controls	One sided *p*-value, independent t-test
General mortality	*Men	49.1	1.34	56.1	1.69	< 0.001
	*Women	55.1	3.16	65.4	4.56	0.035
Suicide mortality	**Men**	41.3	1.81	43.7	3.18	0.222
	**Women**	33.8	3.65	37.5	5.64	0.496

*Statistically significant at the 95% confidence level

In the study, 63 individuals passed away from suicide (suicide, ICD-10 codes X60-X84, being either a contributing or underlying cause of death), 41 (1.2%) of these individuals were cases and 22 (0.3%) controls, with a significantly higher proportion of cases dying from suicide (*p* < 0.001). This difference was statistically significant for men but not for women, Table 1. Suicide death, death of unknown intent and death from respiratory disease were all significantly more common in cases.

The mean age of death from suicide was 40.4 (standard error 1.68) for cases and 44.3 (standard error 2.79) for controls, with no significant age difference (one sided *p* = 0.149). No significant difference was seen when controlling for men and women separately Table 2.

For cases, suicide was the leading underlying cause of death (*n* = 41, 30.9%) followed by neoplasms and diseases of the circulatory system. For controls, neoplasms were the leading underlying cause of death (*n* = 25, 27.8%) followed by diseases of the circulatory system and suicide. In Table 3 all underlying causes of deaths are displayed.

**Table 3:** Underlying cause of death (*N* = 226) amongst cases and controls categorized according to the international classification of disease 10th revision (ICD-10) Chi square comparison or Fischer test (2-sided Fischer significance indicated with^F^) P value adjusted with Benjamin Hochberg correction

ICD-10 code	Disease	*n*	%	Case *n*	Case %	Control *n*	Control %	*P* value	Adjusted *p* value
AB	**Certain infectious and parasitic diseases**	4	1.77	3	2.2	1	1.1	0.105^F^	0.236
CD	**Neoplasms**	44	19.5	19	14.0	25	27.8	0.240	0.393
E	**Endocrine**,** nutritional**,** and metabolic diseases**	4	1.8	3	2.2	1	1.1	0.105^F^	0.210
F	**Mental and behavioral disorders**	6	2.7	4	2.9	2	2.2	0.399^F^	0.552
G	**Diseases of the nervous system**	10	4.4	3	2.2	7	7.8	1.000^F^	1.000
I	**Diseases of the circulatory system**	34	15.0	16	11.8	18	20.0	0.072	0.185
J*	Diseases of the respiratory system*	11	4.9	9	6.6	2	2.2	0.001^F^	0.005
K	**Diseases of the digestive system**	7	3.1	3	2.2	4	4.4	0.690^F^	0.828
M	**Diseases of the musculoskeletal system**	1	0.4	0	0	1	1.1	1.000^F^	1.000
N	**Diseases of the genitourinary system**	3	1.3	1	0.7	2	2.2	0.556^F^	0.715
Q	**Congenital malformations**	2	0.9	1	*0*	2	2.2	1.000^F^	1.000
R	**Unknown cause of death**	11	4.9	7	5.1	4	4.4	0.047^F^	0.141
V0-X59	**Accidents (excluding accidental poisoning)**	9	4.0	5	3.7	4	4.4	0.163^F^	0.293
X40–49*	Accidental poisoning by and exposure to noxious substances*	13	5.8	10	7.4	3	3.3	0.001^F^	0.004
X60–X84*	Suicide*	54	24.3	41	30.9	13	14.4	< 0.001	< 0.001
X85-Y09, Y35-Y36	**Assault**,** legal intervention**,** or operation of war**	1	0.4	1	0.7	0	0	0.326^F^	0.489
Y10–Y34*	Event of undetermined intent*	11	4.9	10	7.4	1	1.1	< 0.001^F^	< 0.001
A–Y*	All deaths*	226	100	136	100	90	100	< 0.001	< 0.001

*Statistically significant at the 95% confidence level after Benjamin Hochberg correction

Suicide was the most common cause of death amongst male cases (*n* = 36, 34.6%) but not female (*n* = 5, 17.2%) for whom death from neoplastic disorders were most common (*n* = 7, 24.1%).

Psychiatric as well as somatic comorbid diseases were abundant in GD, depressive disorders were common with 38% having a depressive comorbid disorder compared to 4.2% of the controls. Alcohol and substance use disorders were also common with a prevalence of 22.8% and 23.1% amongst cases versus 2.8 and 2.5% amongst the controls.

The multifactor Cox regression model did not show a statistical association at the 0.05 alpha level with regards to neither suicide nor general mortality for men nor women. For men low educational level was the factor with strongest effect on as well suicide and general mortality. For women, comorbid respiratory, cardiac, or neoplastic disorder was the factor most strongly associated with general mortality, no significantly associated factors were found with regards to suicidality. The full results are displayed in Table 4 with regards to general mortality and Table 5 for suicide mortality.

**Table 4:** Multivariate Cox regression with general mortality as outcome stratified for sex

Gender	Factor	HR	95 % CI		*P*-value
			Lower	Upper	
Male	**Case vs control**	1.4	0.9	2.1	0.148
	*Age	1.0	1.0	1.0	<0.001
	*ICD CD, Neoplasms, diseases of the blood, and	1.8	1.3	2.6	0.001
	**ICD I, Diseases of the circulatory system**	1.4	1.0	2.0	0.090
	*ICD J, Diseases of the respiratory system	1.6	1.1	2.2	0.009
	*ICD X60–X84, Intentional self-harm	2.2	1.4	3.3	<0.001
	*ICD F10-F19, Substance use disorder	2.4	1.6	3.5	<0.001
	**ICD F32–F33, Depressive disorder**	1.2	0.8	1.8	0.411
	**ICD F40–F48, Anxiety disorder**	0.9	0.6	1.3	0.595
	*Elementary school education vs higher education	7.9	3.5	17.8	<0.001
	**Socioeconomic status**	1.1	0.7	1.5	0.717
Female	**Case (vs control)**	1.6	0.6	4.0	0.315
	*Age	1.1	1.1	1.1	<0.001
	*ICD CD, I or J Neoplasms, Diseases of the circulatory or Diseases of the respiratory system	11.2	2.5	50.2	0.002
	**ICD F, Psychiatric comorbidity**	1.9	0.7	5.5	0.229
	**Socioeconomic status**	0.7	0.3	1.5	0.331

*Statistically significant at the 95% confidence level

**Table 5:** Multivariate Cox regression with suicide mortality as outcome (ICD-10 codes X60-X84 as underlying or contributing cause of death). Stratified for sex

Gender	Factor	HR	95 % CI		*P*-value
			Lower	Upper	
Male	**Case vs control**	2.0	1.0	4.2	0.056
	**Age**	1.0	1.0	1.0	0.128
	*ICD F10-F19, substance use disorder	2.3	1.2	4.5	0.015
	**ICD F32–F33, Depressive disorder**	1.5	0.7	3.1	0.282
	**ICD F40–F48, Anxiety disorder**	1.9	0.9	4.0	0.099
	*Elementary school education vs higher educaton	16.0	2.2	116	0.006
Female	**Case vs control**	1.3	0.2	6.6	0.784
	**Age**	1.0	0.9	1.0	0.269
	**ICD F, Psychiatric comorbidity **	3.3	0.6	19.0	0.179

*Statistically significant at the 95% confidence level

## Discussion

Men and women with GD appear to have an increased mortality compared to age and gender matched controls and decease at a younger age.

The increased mortality rates are in alignment with the notion that mortality rates are elevated in individuals with psychiatric diagnoses. These results confirm and solidify the increased general mortality seen in a previous attempt to outline mortality in GD (Karlsson & Hakansson, 2018). The above-mentioned study is also a Swedish nationwide register study, although with inclusion between 2005 and 2016. Data are thus not entirely comparable, since treatment uptake in the Swedish healthcare sector has increased significantly. Thus, it is possible that individuals diagnosed with GD might have different characteristics than the few diagnosed during the earlier periods, (Hakansson et al., 2018) although many still remain undiagnosed. Further, in 2018, Swedish legislation was updated so that individuals with GD now have the same rights to treatment and aid from the health care and social service sector as individuals with SUD and other psychiatric disorders (Socialstyrelsen, 2017).

Suicide rates amongst individuals with GD were also markedly higher confirming the increased suicide deaths seen in the previous nationwide register study (Karlsson & Hakansson, 2018). GD did not remain a significant risk factor at the 0.05 alpha level in the adjusted analysis. The relatively small number of suicide deaths implicates that these results should be interpreted cautiously but it is likely that low education level and substance use disorders are stronger risk factors for suicide. Further studies are needed to investigate the impact of GD on suicide mortality especially for women.

Although GD did not appear as an independent risk factor at the 0.05 alpha level of significance there was a tendency towards a risk increase associated with mortality as well as suicide mortality for both men and women and it is apparent that individuals with GD are at increased risk of premature death as well as suicide death. Somatic disorders and respiratory disorders especially, and thus likely smoking should be screened for in order to prevent premature general mortality in GD.

### Limitations

It is important to keep in mind that a smaller fraction of those with gambling disorder are diagnosed in national healthcare, and that there are many barriers to treatment seeking (Gainsbury et al., 2014). It is intuitive to think that individuals who, despite this, are diagnosed in the specialist health care system represent a sub-population with more comorbidity and a worse addiction. Further, it is plausible that individuals treated only within the social services might to those diagnosed in health care. As social services might be increasingly involved in the care of GD due to recent legislative changes in Sweden rates of social welfare payments might be changing which might impact suicidality. These relationships need further investigation although beyond the scope of the present study.

This study did not explore the temporal relationships between GD and comorbid psychiatric diagnosis. This is a delicate matter in register research; however, it is relatively easy to identify which diagnosis was given at a certain time it is possible that GD is less screened for and more lately diagnosed than conditions which the health care have longer experience of treating and more knowledge on. Thus, in this study we have chosen to not investigate temporal relationships as it was not our main focus.

Further, a statistical limitation is the potential bias of competing risk with regards to suicide mortality. An individual might indeed pas away due to somatic illness who otherwise would pass away due to suicide. In our Cox regression that individual would be censored at time of death thus only contributing with time until death, but no further adjustments with regards to ‘competing risks’ were made. In addition, while our research employed a hypothesis-driven selection of variables and explanatory modeling, we recognize that alternative data-driven methods, such as penalized regression techniques (e.g., Lasso, Elastic Net), might offer complementary insights by uncovering additional predictors or improving model specification. Future studies with larger sample sizes and more events should consider applying these methods to validate and build upon our results.

### Strengths

This is, to the best of the authors’ knowledge, the first case-control study to investigate mortality and suicide mortality in GD. Matching individuals with GD to individuals of the same gender, age and from the same municipality implies that mortality and suicide levels can be compared quite precisely without any interference of gender and age, and minimizing differences in living conditions such as living in an urbanized area or not. Further, these registers provided unique opportunity to examine the effects of socioeconomic risk-factors and comorbid disorders. It is also a nationwide study with a decent number of participants and an average follow-up of almost five years.

## Conclusion

Individuals with gambling disorder had an increase in levels of mortality and suicide mortality compared to age, gender and municipality-matched controls. However, gambling disorder itself was not at the 0.05 alpha-level statistically associated with neither suicide nor general mortality when controlling for somatic and psychiatric comorbidities, gender, age and socioeconomic status. Thus individuals with gambling disorder suffer from increased mortality and suicide mortality and reasons for these appear to be multifactorial motivating careful suicide risk assessment and screening for somatic comorbidities in individuals with gambling disorder.

## Data Availability

Due to risk of indirect identification, data cannot be shared outside of the research group in accordance with our ethical permit and Swedish legislation.
